# Pacemaker detected active minutes are superior to pedometer-based step counts in measuring the response to physical activity counseling in sedentary older adults

**DOI:** 10.1186/s12877-020-01559-y

**Published:** 2020-05-06

**Authors:** Venkata K. Puppala, Benjamin C. Hofeld, Amberly Anger, Sudhi Tyagi, Scott J. Strath, Judith Fox, Marcie G. Berger, Kwang Woo Ahn, Michael E. Widlansky

**Affiliations:** 1 Division of Cardiovascular Medicine, Department of Medicine, Medical College of Wisconsin, Milwaukee, WI USA; 2 Department of Medicine, Medical College of Wisconsin, Milwaukee, WI USA; 3 College of Health Sciences Department of Kinesiology, University of Wisconsin-Milwaukee, Milwaukee, WI USA; 4 Department of Biostatistics, Medical College of Wisconsin, Milwaukee, WI USA; 5 Department of Pharmacology, Division of Cardiovascular Medicine, Medical College of Wisconsin, Hub for Collaborative Medicine 5th Floor 8701 W Watertown Plank Road, Milwaukee, WI USA

**Keywords:** Pacemaker, Physical activity, Accelerometer, Cardiology, Geriatrics

## Abstract

**Background:**

In patients with permanent pacemakers (PPM), physical activity (PA) can be monitored using embedded accelerometers to measure pacemaker detected active hours (PDAH), a strong predictor of mortality. We examined the impact of a PA Counseling (PAC) intervention on increasing activity as measured by PDAH and daily step counts.

**Methods:**

Thirteen patients (average age 80 ± 6 years, 84.6% women) with implanted Medtronic PPMs with a ≤ 2 PDAH daily average were included in this study. Patients were randomized to Usual Care (UC, *N* = 6) or a Physical Activity Counseling Intervention (PACI, *N* = 7) groups. Step count and PDAH data were obtained at baseline, following a 12-week intervention, then 12 weeks after intervention completion. Data were analyzed using independent t-tests, Pearson’s r, chi-square, and general linear models for repeated measures.

**Results:**

PDAH significantly differed by time point for all subject combined (*P* = 0.01) but not by study group. Subjects with baseline gait speeds of > 0.8 m/sec were responsible for the increases in PDAH observed. Step counts did not differ over time in the entire cohort or by study group. Step count and PDAH significantly correlated at baseline (*r* = 0.60, *P* = 0.03). This correlation disappeared by week 12.

**Conclusion(s):**

PDAH can be used to monitor PA and PA interventions and may be superior to hip-worn pedometers in detecting activity. A significant increase in PA, regardless of treatment group, suggests that patient awareness of the ability to monitor PA through a PPM increases PA in these patients, particularly in patients with gait speeds of < 0.8 m/sec.

**Trial registration:**

ClincalTrials.gov NCT03052829. Date of Registration: 2/14/2017.

## Background

The benefits of habitual physical activity [PA], activities of at least moderate intensity defined as ≥3 metabolic equivalents (METs)], are well-recognized. Emerging information from large data sets strongly suggests high levels of sedentary behavior, defined as activities < 1.5 METs (e.g. seated activities such as computer work) increases the risk of diabetes, cardiovascular disease, and death, independent of the amount and intensity of PA [1–5]. Morbidity and mortality associated with non-adherence to PA per guidelines established by US Department of Health and Human services (DHHS) is estimated at $117 billion annually [6–8]. The increased risk of sedentary behavior appears to be mediated at least in part by reduced insulin sensitivity, impaired lipid metabolism, increased vascular inflammation, and increased thrombotic tendencies [9–14]. Aging is associated with sedentary behavior and only 25% of the adults aged > 50 years are able to achieve PA goals per DHHS guidelines [15]. Patients with permanent pacemakers (PPM) can be a target population for risk modification strategies to increase the PA levels. Pacemaker recipients are typically older [16]. Demographic trends show that the average age of PPM recipients is increasing with greatest increase seen in the rate of placements in those ages 75 and above [17]. Pacemaker recipients also have a higher prevalence of coronary artery disease (CAD) [18, 19]. Physical activity counseling (PAC) can be used as an effective strategy to increase activity level and reduce the risk of morbidity and mortality associated with chronic diseases [20]. Feedback mechanisms using devices like pedometers and accelerometers can be useful for tracking physical activity quantity and intensity as well as motivating patients to increase their activity levels [21].

The internal accelerometer embedded in Medtronic pacemakers registers, stores, and reports total “active time” based on a threshold activity intensity level of approximately 70 steps/min (estimated to be > 1.5 METs). The accelerometers in the pacemakers are useful for sensing the activity level and facilitate adaptive rate responsiveness to meet the physiological demands of the patient [22, 23]. This implanted accelerometer, combined with the regular follow-up required appropriate changes in the pacemaker settings in these individuals, provides an excellent opportunity to determine the impact of sedentary behavior on mortality and cardiovascular events. We recently reviewed the medical records of 96 individuals who underwent de novo Medtronic EnRhythm™ PPM implantation for sinus nodal dysfunction or complete heart block.^13^ Following a 6-month blanking period post implantation to allow for patient acclimation to their PPM and early programming changes, accelerometer data obtained from interrogations were abstracted and averaged over a 1-year period. Individuals were categorized as having failed to reach (*n* = 40) or having met/exceeded (*n* = 54) the activity threshold for ≥2 h/day. Of those who failed to achieve the activity threshold for ≥2 h/day, 35% died (14/40) vs. 7.4% (4/54) of the group who achieved this threshold. Survival analyses demonstrated a significantly greater mortality for those with increasing sedentary time (*P* < 0.001 by log rank test). Following adjustment of sex, prevalent CAD, and LVEF < 50%, low activity remained a significant risk factor for death.

Overall, these data suggest an easy to implement, point-of-care PA intervention designed to reduce inactive time as measured by Medtronic pacemaker accelerometer data, that could potentially reduce risk in patients with implanted permanent pacemakers. However, prior to a larger, outcomes-based study, there is a need to establish that active time, as measured by the pacemaker accelerometer, tracks changes in PA with an intervention. Our pilot study tested whether a point of care method that combines informing at-risk patients of our published findings and their own active time amounts, combined with an intervention to increase moderate intensity activity in daily living will result in detectable increases in pacemaker-measured active minutes. We compared these findings to physical activity as measured by an externally worn pedometer, a commonly used tool for measuring physical activity in clinical studies.

## Methods

### Subject recruitment

**Fig. 1:**
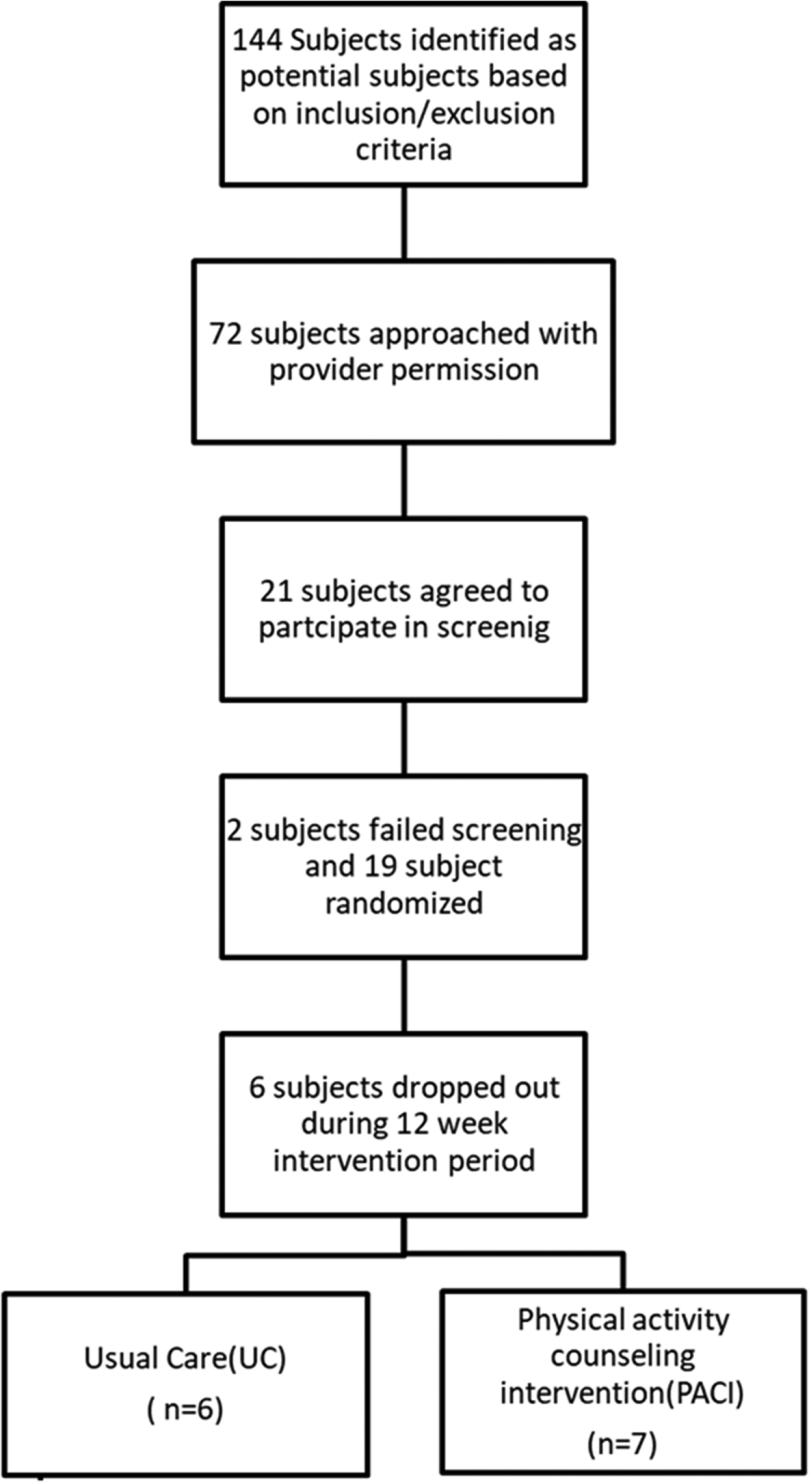
Recruitment and Study Participation Flow Chart

All study procedures were reviewed and approved by the Medical College of Wisconsin’s Institutional Research Board. Under a HIPAA waiver of authorization, medical records from individuals attending Froedtert and Medical College of Wisconsin’s Electrophysiology Clinic were screened for potential enrollment. Figure 1 illustrates study enrollment. The electrophysiology providers for subjects meeting inclusion and exclusion criteria were contacted to introduce the study to the potential subjects and obtain approval for the study team to contact the potential subject for further details. Study inclusion criteria included the following: age > 55 years, presence of a Medtronic Azure, Advisa, Revo, or EnRhythm PPM (to ensure in-device accelerometers reporting pacemaker detected active hours (PDAH) with an identical algorithm), ability to ambulate 650 steps over 10 min, LVEF ≥50% on their most recent echocardiogram, and an average PDAH of ≤2 h over the three-month period prior to enrollment (as estimated from the 12-month graphical output from the device interrogation as previously reported) [24]. Subjects were excluded if they had a life expectancy of less than 1 year at the time of enrollment/implantation, known history of cognitive impairment or inability to follow study procedures, or post-pacemaker implantation follow-up at a non-study center.

### Study procedures

#### Screening, randomization, and intervention

The study screening visit included a detailed medical history, including a medication history. Potential subjects had their height and weight measured and their heart rate and blood pressure measured in triplicate and averaged. A walk test was administered to assure the potential subject could walk well enough (at least 650 steps in 10 min) to be included in the study. Gait speed for each subject was calculated by using the amount of time it took for a subject to take 650 steps and using published age- and sex-specific normative values for step length for older adults [25].

Individuals passing the screening visit were subsequently randomized to either a physical activity counseling intervention (PAC) or usual care (UC). The PAC employed a 5 A’s (Assess, Advise, Agree, Assist, Arrange) model previously reported to be effective for other health behaviors [26]. Subjects randomized to the PAC arm met immediately following randomization with an intervention expert on the study staff and advised of their activity levels. This included a review of the average active hours over the past 3 months along with a review of our previously reported data on the association of lower pacemaker-measured active time with increased mortality [24]. PAC subjects migrated through the 5 A’s intervention model for 12 weeks. The intervention began with an initial educational visit. This visit consisted instruction on the use of objective, uploadable enhanced stepcount monitors (Omron HJ-112, Kyoto, Japan), and, access and use of the specially designed web-mediated individually tailored physical activity and health web platform. The web platform leverages strategies (frequent feedback, realistic goal-setting, rewarding, and self-regulation) proven to successfully integrate of lifestyle-based physical activity into the daily lives of older adults [27–31]. PAC subjects were also sent weekly information on cognitive and behavioral strategies to increase health enhancing lifestyle practices and physical activity through the interactive website. Generally, the intervention was designed to encourage PAC subjects to increase steps by 10% per week as measured by their daily pedometer-based step counts which they record on a calendar supplied to them. The weekly web-mediated interactions were phased. Phase 1 (weeks 1–6) was designed to provide a cognitive understanding of the benefits associated with physical activity, current physical activity recommendations that are associated with healthful behaviors, and objective self-awareness of their current physical activity levels, obtained through body worn uploaded step count data. Phase 2 (weeks 6–12), continued to build upon educational information, and required each subject to intrinsically set daily physical activity goals for themselves. On a weekly basis, subjects uploaded their physical activity information and were subsequently provided with graphical representations of daily steps and how such values correspond with intrinsically set goals. At this stage of the program, each subject was either in compliance with set goals (defined as meeting physical activity goal targets 5 out of 7 days), or they were not in compliance. Subjects who successfully achieved their weekly goal were congratulated by the software and given guidance for setting the goals for the ensuing weeks. If the subject failed to reach their goal, the software attempted to ascertain the barriers associated with the inability to reach goal and gave pre-specified motivational messages offers strategies for succeeding based on identified barriers. In addition, PAC subjects received bi-weekly telephone check-in carried out by a trained behavioralist who reviewed their activity and offered support, advice, and solicited feedback. The 12 week length of the intervention period was selected as this is the period of time has been previously established to demonstrate favorable effects on vascular structure and function known to be associated with favorable reductions in cardiovascular risk in older adults [32–34].

At the end of the 12-week active intervention, PAC subjects entered a 12-week maintenance phase during which time no calls were made to the subjects by the study team nor was any information on increasing physical activity shared with the subjects by the study team. A twelve-week maintenance period was chosen to allow for the study team to determine near term ability of the intervention to increase activity levels in this at-risk population. Subjects in the UC arm did not receive any intervention. They were mailed a pedometer and a step count calendar to record their steps for the one-week periods corresponding to the beginning of the study, the 12th week in the study, and the 24th week in the study (time points corresponding to the start and end of the PAC groups intervention and maintenance periods).

#### Pacemaker derived active hours (PDAH) extraction

PDAH was obtained from pacemaker interrogations performed during clinical visits or trans-telephonic transmission of pacemaker information, corresponding to weeks 1, 12, and 24. PDAH was calculated as an average of the daily active hour time over the 3-month period prior to each measurement timepoint estimated as previously described and validated.^13^

#### Statistical analyses

SPSS 24 and SigmaStat 12.5 were employed for data analyses. Data were analyzed on a per protocol basis given that the goal of this pilot study was to determine how well PDAH tracked activity over time rather than PACI efficacy. Baseline characteristics were compared between groups using unpaired t-tests, chi-square, or Fisher’s Exact test as appropriate based on variable type and number of events. Differences in step counts and PDAH over the study period were investigated using general linear models for repeated measures with the randomization group assignment as the between subjects variable and a three factor within subjects comparisons representing the three measurement time points with the Tukey test applied for post-hoc comparisons if significance of the overall models was detected. Additional analyses were carried out comparing those with a gait speed above versus below 0.8 m/sec at baseline, a gait speed cut-off associated with overall frailty and increased mortality [35, 36]. Correlations between step count measurements and PDAH measurements were performed using Pearson’s r test. *P* < 0.05 were considered significant.

## Results

### Subject demographics

Overall enrollment data are summarized in Fig. 1. The study was initially designed to enroll 30 subjects but was stopped secondary to challenges with enrollment. One hundred forty-four individuals fit our inclusion and exclusion criteria based on IRB-compliant pre-screening of the electronic medical record. We were given permission to approach 72 potential subjects about the study by their providers. Of these subjects, a total of 21 subject agreed to study screening. Two subjects failed screening and were not enrolled. Six subjects dropped out following randomization due to health issues for them or their significant others not related to the study protocol, leaving a total of 13 subjects (*N* = 7 in the PDAH arm, *N* = 6 in the UC arm) who completed the study protocol. Subject characteristics for the entire study cohort and the cohort by randomized study group are presented In Table 1. The UC group was significantly younger than the PDAH group (*P* = 0.01), but otherwise overall attributes were roughly similar despite small numbers. While the left ventricular ejection fraction was statistically significantly lower in the UC group than the PDAH group (*P* = 0.02), the average left ventricular ejection fractions were within the normal range in both groups. There were no significant differences between groups with respect to calculated gait speed (*P* = 0.85).

**Table 1:** Patient baseline demographics

	All Subjects (*n* = 13)	PAC (*n* = 7)	UC (*n* = 6)	*P* value (UC vs PAC)
Female sex- N (%)	11 (84.6)	6 (85.6)	5 (83.3)	1.00
Age (yrs)	80 ± 6	84 ± 5	75 ± 5	0.01*
BMI (kg/m^2^)	31.2 ± 5.7	30.0 ± 5.0	32.5 ± 6.6	0.46
Ethnicity				1.00
*Black (%)*	2 (15.4)	1 (14.3)	1 (16.7)	
*White (%)*	11 (84.6)	6 (85.7)	5 (83.3)	
Indication for PPM Placement				0.27
Sick Sinus Syndrome	4 (30.8)	1 (14.3)	3 (50)	
High Degree AV Block	9 (69.2)	6 (85.7)	3 (50)	
History of Sustained Ventricular Tachycardia N(%)	3 (23.1)	0 (0)	3 (50)	0.84
History of Non-Sustained Ventricular Tachycardia N(%)	7 (53.8)	2 (28.6)	5 (71.4)	0.10
Creatinine Clearance < 60 mL/min/1.73 m^2^ N(%)	5 (38.5)	4 (57.1)	1 (16.7)	0.26
Hypertension N(%)	10 (76.9)	6 (85.7)	4 (66.7)	0.56
Hyperlipidemia N (%)	12 (92.3)	6 (85.7)	6 (100%)	1.00
Diabetes N (%)	3 (23.1)	0 (0)	3 (50%)	0.07
Heart Failure N (%)	2 (15.4)	0 (0)	2 (33.3%)	0.19
Myocardial Infarction N(%)	1 (7.7)	0 (0)	1 (16.7)	0.46
Coronary Stent N(%)	2 (11.77)	1 (14.3)	0 (0)	1.00
Prior CABG N(%)	2 (15.4)	1 (14.3)	1 (16.7)	1.00
Prior CVA N (%)	5 (38.5)	1 (14.3)	4 (80.0)	0.10
Atrial Fibrillation (%)	7 (53.8)	3 (42.9)	4 (57.1)	
LVEF %	60 ± 4	62 ± 3	57 ± 4	0.02*
LV EDD (cm)	44 ± 5	43 ± 3	44 ± 6	0.62
LV ESD (cm)	28 ± 6	26 ± 2	30 ± 9	0.25
Percent Atrial Pacing	61 ± 34	66 ± 31	53 ± 39	0.50
Percent Ventricular Pacing	22 ± 38	12 ± 21	34 ± 51	0.34
Mitral Regurgitation – Moderate or more	2 (15.3)	2 (28.6)	0 (0)	0.46
Current Smoker (%)	1 (7.7)	0 (0)	1 (16.7)	0.46
ACE Inhibitor or ARB Therapy (%)	7 (53.8)	3 (42.9)	4 (57.1)	0.59
Beta Blocker Therapy (%)	7 (53.8)	4 (57.1)	3 (50)	1.00
HMG CoA-Reductase Therapy (%)	8 (61.5)	4 (57.1)	4 (66.7)	1.00
SBP (mmHg)	126 ± 22	131 ± 27	121 ± 14	0.44
DBP (mmHg)	73 ± 114	70 ± 9	77 ± 18	0.38
HR (bpm)	68 ± 6	66 ± 4	70 ± 7	0.18
Calculated Gait Speed (m/sec)	0.85 ± 0.16	0.84 ± 0.14	0.86 ± 0.18	0.85

Values are reported in mean (SD) or as absolute numbers with (% of n). *P* values compare baseline demographics of UC and PAC groups.*P < 0.05

### Results of the intervention

**Fig. 2:**
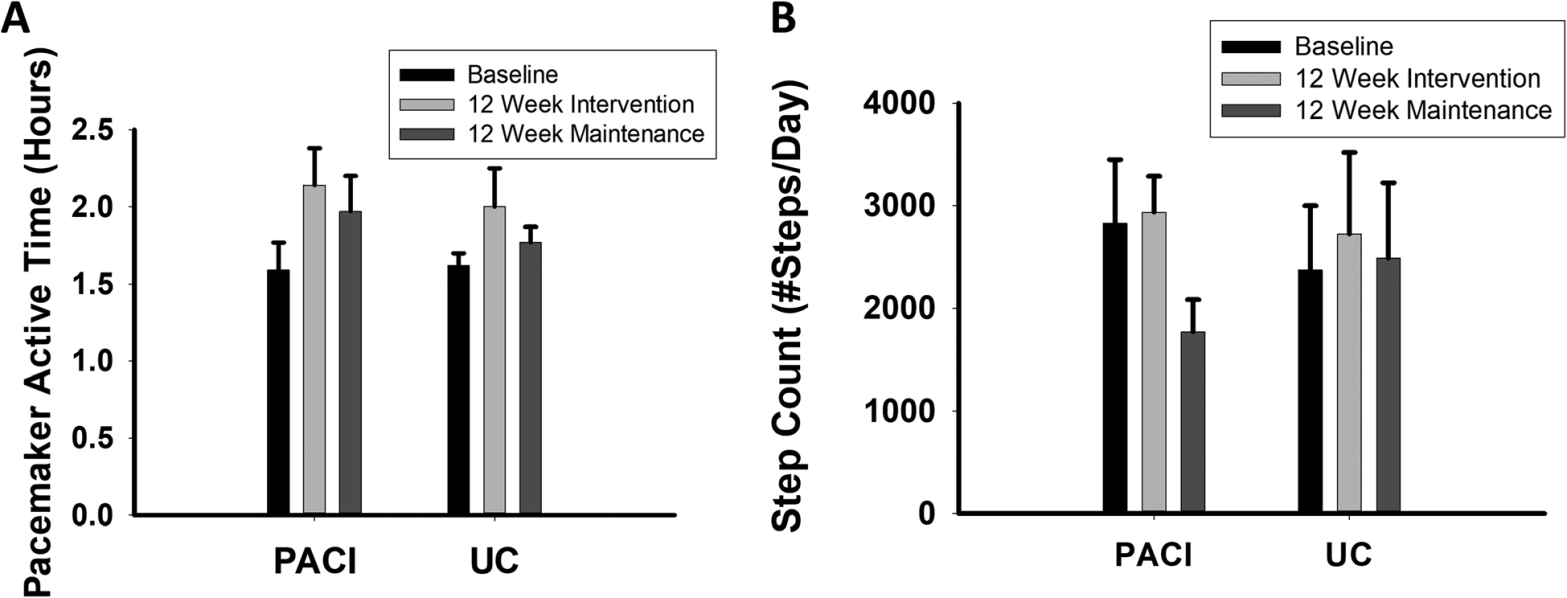
PDAH and Step Count Results for each study period. **a** PDAH results showed an overall increase in PDAH comparing the 12-week intervention period to baseline that did not significantly differ between the PACI and UC arms. **b** Step Count results showed no differences in measured step counts over the time course of the study and no differences in step counts between the PACI and UC groups. See Table 2 and the results section for additional details. PDAH- Pacemaker-Derived Active Hours. PACI- Physical Activity Counseling Intervention. UC- Usual Care. Data presented as mean (SEM)

PDAH and step counts as recorded by the study subjects for weeks 1, 12, and 24 of the study periods are presented in Tables 2, 3, and Fig. 2.

**Table 2:** PDAH Results by Study Group

	Pre-Intervention (average hours of active time/day)	Post-12 Week Intervention (average hours of active time/day)	Post-12 Week Maintenance Period (Week 24) (average hours of active time/day)
**PAC Group (*n*** **= 7)**	1.59 ± 0.44	2.14 ± 0.61	1.97 ± 0.56
**UC Group (*n*** **= 6)**	1.62 ± 0.18	2.00 ± 0.56	1.77 ± 0.22

*PDAH* pacemaker derived active hours

**Table 3:** Pedometer-Based Step Count Results by Study Group

	Pre-Intervention (average daily step count)	Post-12 Week Intervention^a^ (average daily step count)	Post-12 Week Maintenance Period (Week 24)^a^ (average daily step count)
**PAC Group (*n*** **= 7)**	2827 ± 1641	2934 ± 786	1769 ± 771
**UC Group (*n*** **= 6)**	2474 ± 1623	2724 ± 1945	2487 ± 1800

^a^Two individuals in the PAC group did not turn in post-12-week intervention step count logs and (*n* = 5 for PAC at that time point). One individual in the PAC group did not turn in post-12-week maintenance period step count logs (*n* = 6 for PAC group at that time point). Data presented as mean (SD). PDAH-pacemaker derived active hours

Over the 12-week intervention period, PDAH increased by 35.1% in the PACI group and 32.5% in the UC group. PDAH significantly increased over time, and this increase did not significantly differ between groups (*P* = 0.01 for time, *P* = 0.69 for time x study group interaction). Post-hoc analyses determined that the combined study groups had significantly greater PDAH during the 12-week interventional study period than during the three-month period prior to beginning the intervention (*P* = 0.005). No significant differences were seen between the pre-study period and the three-month maintenance phase (*P* = 0.15). There was a trend toward a decrease in PDAH during the three-month maintenance phase compared to the 12-week intervention period (*P* = 0.052).

No differences were seen between any time point by step count (*P* = 0.08 for time, *P* = 0.19 for time x study group interaction). Multiple imputation techniques were used to account for the missing step count data points (two individuals in the PAC group did not turn in week 12 step count data and one individual in the PAC group did not turn in week 24 step count data). The data analyzed following multiple imputation did not differ from the raw data (data not shown).

Three subjects in the PAC group and one subject in the UC group used walking assist devices at least intermittently. The average gait speed of these subjects was not significantly different than those who did not use any assist devices (0.89 ± 0.10 vs. 0.84 ± 0.18 m/sec, *P* = 0.57). The results for these subjects was not significantly different from those who did not use assist devices in the pattern of activity time and step counts throughout the study and did not affect the overall results (data not shown).

### Analysis of Results Based on Gait Speed

To determine whether baseline gait speed impacted our results, we stratified our study cohort into two groups with a cut-off gait speed of 0.8 m/sec. Three subjects in the PAC group and two subjects in the UC group had baseline gait speeds ≤0.8 m/sec. There was a significant change in PDAH over time (*P* = 0.01) with a significant interaction between time and gait speed (*P* = 0.02). As shown in Table 4, those subjects with baseline gait speeds > 0.8 m/sec showed significant improvements in PDAH at the end of the 12 week intervention period which remained improved 12 weeks following the cessation of the intervention. Subjects with a baseline gait speed of > 0.8 m/sec significantly increased PDAH from baseline by week 12 of the intervention (*P* < 0.001) and maintained that increase 12 weeks following the end of the intervention phase (*P* = 0.01). There was no significant drop in PDAH between weeks 12 and 24 in those with baseline gait speed > 0.8 m/sec (*P* = 0.16). PDAH was significantly higher in those with gait speed > 0.8 m/sec compared to those ≤0.8 m/sec at week 12 (*P* = 0.007) and there was a strong trend toward greater PDAH at week 24 in those in the faster gait speed group (*P* = 0.06). No changes were observed over the 24-week study period in those in the lower gait speed group (*P* > 0.94 for all comparisons of PDAH between all 3 time points in the lower gait speed group).

**Table 4:** PDAH Results by Gait Speed Category

	Pre-Intervention (average hours of active time/day)	Post-12 Week Intervention (average hours of active time/day)	Post-12 Week Maintenance Period (Week 24) (average hours of active time/day)
**> 0.8 m/sec (*n*** **= 8)**	1.57 ± 0.37	2.33 ± 0.48*	2.05 ± 0.48^*^
**≤ 0.8 m/sec (*n*** **= 5)**	1.64 ± 0.33	1.67 ± 0.45^†^	1.88 ± 0.43

*P < 0.05 vs. Pre-Intervention in those with gait speed > 0.8 m/sec, ^†^*P* < 0.05 vs. gait speed > 0.8 m/sec at the same study time point. Data presented as mean (SD). PDAH- pacemaker derived active hours

Similar differences were not detected by pedometer-based step counts (*P* = 0.23 for changes in step counts over time, *P* = 0.93 for time/gait speed interaction, Table 5). Re-analysis of the PDAH data by study group (PAC vs. UC, Table 6) showed a pattern similar to the overall study with a significant increase PDAH with time regardless of study group (*P* = 0.002 overall, *P* = 0.70 for study group/time interaction).

**Table 5:** Pedometer-Based Step Count Results by Gait Speed Category

	Pre-Intervention (average daily step count)	Post-12 Week Intervention (average daily step count)	Post-12 Week Maintenance Period (Week 24) (average daily step count)
**> 0.8 m/sec (*n*** **= 6)**	2988 ± 11,319	3238 ± 1510	2650 ± 1636
**≤ 0.8 m/sec (*n*** **= 4)**	1718 ± 1080	1852 ± 1077	1476 ± 1018

Data presented as mean (SD)

**Table 6:** PDAH Results -Subjects with Gait Speed > 0.8 m/sec Only

	Pre-Intervention (average hours of active time/day)	Post-12 Week Intervention (average hours of active time/day)	Post-12 Week Maintenance Period (Week 24) (average hours of active time/day)
**PAC (*n*** **= 4)**	1.48 ± 0.50	2.48 ± 0.61	2.20 ± 0.68
**UC (*n*** **= 4)**	1.68 ± 0.21	2.20 ± 0.35	1.90 ± 0.08

Data presented as mean (SD). *PDAH* pacemaker derived active hours, *PAC* Patient Activity Counseling, *UC* Usual Care

### Correlations between step counts and PDAH

The correlations between step counts and PDAH count at each study time point are presented in Table 7.

**Table 7:** Correlations between step counts and PDAH throughout the study

	Pre-Intervention	Post-12 Week Intervention	Post-12 Week Maintenance Period (Week 24)
**Correlation Coefficient (r)**	0.6	0.17	−0.04
***P*-Value**	0.03*	0.96	0.90

*PDAH* Pacemaker-derived activity hours

* *P*-Value < 0.05

While PDAH correlated reasonably well with step count prior to beginning the interventional study (*r* = 0.60, *P* = 0.03), PDAH did not correlate with step counts at the week 12 and week 24 timepoints (*r* = 0.17, *P* = 0.96 and *r* = − 0.04, *P* = 0.90 for week 12 and 24 timepoints, respectively). Given the significant correlation between step count and PDAH prior to the intervention, we performed additional correlations between PDAH at this time point and clinical variables including age (*r* = 0.36, *P* = 0.24), systolic blood pressure (*r* = 0.53, *P* = 0.06), diastolic blood pressure (*r* = 0.36, *P* = 0.23), LV ejection fraction (*r* = − 0.10, *P* = 0.75), LV end-systolic dimension (*r* = 0.04, *P* = 0.91), and LV end-diastolic dimension (*r* = − 0.02, *P* = 0.94). None of these measured significantly correlated with PDAH.

## Discussion

In this small pilot study, we found that PDAH was able to capture increases in activity levels with enrollment and participation in the study, and that these increases were independent of the intervention in this study. While numerically still low, the observed increases were significant with activity levels increasing by approximately 33% from baseline in each study arm. We were unable to visualize any significant changes in physical activity over the study period using externally worn pedometer captured step counts. As discussed below, the reasons for this discrepancy is likely multifactorial and driven by the unique challenges to accurate step counts by pedometer of our older study population (average age 80 ± 6 years) as well as study subjects participating in activities in which wearing a pedometer was not feasible. The finding of no correlation between step counts and PDAH following the intervention and maintenance periods supports the hypothesis that the increase in PDAH was not detected by step count measurements. In addition, we found that participating in the study resulted in significant increases in PDAH in only those with a baseline gait speed > 0.8 m/sec. Overall, these data suggest that implanted pacemakers with embedded accelerometers can track increases in activity levels in this challenging study population, and may do so more reliably than externally worn pedometers. In addition, the data suggest physical activity levels in this population can be influenced by provider attention to overall physical activity, particularly in those with a gait speed > 0.8 m/s.

Prior work from our group demonstrates that PDAH is a strong predictor of mortality in patients with pacemakers [24]. An increased prevalence and severity of frailty has also been associated with reduced activity levels as measured by implanted cardiac devices including pacemakers [37]. These data from pacemaker-based studies are consistent with prior work in those with reduced left ventricular ejection fractions evaluating devices placed for resynchronization and/or protection from lethal arrhythmia [38]. Our data extend these findings by demonstrating that an intervention designed to increase physical activity can also be detected by implanted pacemakers, which may be superior to externally worn devices given their lack of susceptibility to human error and reduced sensitivity of external pedometers at low gait speeds (more common in this population) for detection of steps and activity [39–41]. These advantages of an implanted device add to the significant advantages of device-quantified PA including its ability to monitor circadian rhythms and better characterize PA architecture and intensity than self-reported activity levels even in older adults [42–44].

We found that physical activity as detected by PDAH increased during the 12-week intervention group regardless of study arm. This suggests our study cohort’s activity levels may have been influenced in part by having a care provider pay attention to their activity level. The increase in activity, while not detected by the external pedometers, may still also be an effective mechanism for feedback to patients to increase activity levels. Our findings also suggest that the PACI could potentially be improved with better tailoring of the intervention to the target population. Some subjects reported that the amount of walking suggested by the intervention was not feasible based on their orthopedic concerns and preferred activities such as swimming for these reasons. Others voiced concerns that the locations suggested by the PACI (e.g. local malls, sports centers, gyms) were too difficult to travel to on a regular basis, particularly during inclement weather. These issues may be in part unique to the older population in this study relative to other physical activity studies and suggest the superiority of implanted devices in tracking the types physical activities in which this older population engages.

Interestingly, we found that participation in the study lead to significant increases in activity levels almost exclusively in those with a gait speed > 0.8 m/sec. Gait speed is well-known as a powerful predictor of mortality with lower gait speeds associated with increased frailty [35, 36]. The threshold of 0.8 m/sec we selected has been shown in multiple studies to stratify mortality risk [35, 36]. A gait speed of 0.8 m/sec is associated with median life expectancy for both men and women with gait speeds ≥1.0 m/sec as consistently associated with better than median survival [35, 36]. Our data suggest targeting those with PDAH under 2 h per day for activity interventions are likely to be most effective in those with a gait speed > 0.8 m/sec while those with slower gait speed may need other interventions to improve strength and/or mobility prior to attempts to increasing overall active time.

A major challenge with the current study was recruitment and subject retention. In screening and discussing the study with potential subjects, we found the average age of individuals with implanted pacemakers and preserved left ventricular ejection fractions averaging less than 2 active hours per day was approximately 80 years of age. Those who were contacted and declined to enroll commonly cited their personal health issues, lack of time, need to care for another family member, already having too many appointments to track, or lack of self-efficacy regarding increasing their activity levels. The six individuals who dropped out of the study following randomization cited new unrelated health issues or feeling overwhelmed with logging steps and participating in study activities. For future projects of this nature working with similar age-groups and populations, these issues merit significant consideration to increase enrollment and retention of study subjects.

This study has some limitations. Our study has a small sample size. However, this study was designed a priori as a pilot study for feasibility and this study achieved its goal with 80% power to detect a difference in PDAH in the intervention group the size of that detect in this study with a standard deviation of 0.45 at α = 0.05. While these data demonstrate that changes in physical activity can be tracked by the specific pacemakers specified in our enrollment criteria, we cannot yet generalize these findings to other pacemakers with different accelerometer algorithms. In addition, whether improvements in PDAH are associated with reduced future adverse cardiovascular events and/or mortality remains unknown. We also cannot determine whether increases in activity seen following the full 24 weeks (including 12 weeks of time without active intervention in the PAC group) would be sustained over longer periods of time. Balanced against these limitations are the unique study population enrolled in this study and the novel findings that in this population PDAH appears superior to pedometer measurements in quantifying improvements in physical activity and that paying attention to physical activity levels in those with relatively preserved gait speed can result in significant improvements in activity levels. Given the critical importance of maintaining physical activity in older adults to preserve and enhance muscle strength, mental acuity, and physical health, these data may particularly helpful in encouraging and monitoring PA on older adults with implanted devices [45–48].

## Conclusions

Overall, we found that a 12-week intervention to increase physical activity could increase activity based on pacemaker-based accelerometer measurements by approximately one-third. In addition, this amount of increase occurred regardless of the intensity of intervention suggesting that increased attention to physical activity in this patient population could lead to increased physical activity. Our findings also suggest that further tailoring a physical activity intervention for the type of study population enrolled in this study as well as targeting sedentary patients with gait speeds > 0.8 m/sec for physical activity interventions may greater benefits than seen this study. PDAH also appears superior pedometer-based step counts to measure changes in activity in this study population, likely due to the multiple reasons previously cited related to subject specific characteristics unique to older adults. Further work will be necessary to best delineate how to encourage increasing physical activity in this population and also to improve methods for subject recruitment and retention prior to larger studies looking at the efficacy of following PDAH to reduce mortality.

## Data Availability

The datasets used and/or analysed during the current study are available from the corresponding author on reasonable request.

## Abbreviations

CAD: Coronary artery disease
PA: Physical activity
PACI: Physical activity counseling intervention
PDAH: Pacemaker derived active hours
UC: Usual care
